# A Simulation Study of the Radiation-Induced Bystander Effect: Modeling with Stochastically Defined Signal Reemission

**DOI:** 10.1155/2012/389095

**Published:** 2012-11-11

**Authors:** Kohei Sasaki, Kosuke Wakui, Kaori Tsutsumi, Akio Itoh, Hiroyuki Date

**Affiliations:** ^1^Graduate School of Engineering, Kyoto University, Kyoto 606-8501, Japan; ^2^Graduate School of Health Sciences, Hokkaido University, Sapporo 060-0812, Japan; ^3^Faculty of Health Sciences, Hokkaido University, Sapporo 060-0812, Japan

## Abstract

The radiation-induced bystander effect (RIBE) has been experimentally observed for different types of radiation, cell types, and cell culture conditions. However, the behavior of signal transmission between unirradiated and irradiated cells is not well known. In this study, we have developed a new model for RIBE based on the diffusion of soluble factors in cell cultures using a Monte Carlo technique. The model involves the signal emission probability from bystander cells following Poisson statistics. Simulations with this model show that the spatial configuration of the bystander cells agrees well with that of corresponding experiments, where the optimal emission probability is estimated through a large number of simulation runs. It was suggested that the most likely probability falls within 0.63–0.92 for mean number of the emission signals ranging from 1.0 to 2.5.

## 1. Introduction

 The radiation-induced bystander effect (RIBE) was initially reported by Nagasawa and Little [1]. Since then, this phenomenon has been observed for different radiation types such as *α* particles, photons, and carbon beams [2–5], and new experimental techniques have been applied to investigate the effect and associated processes [6]. Particularly, the microbeam technique has demonstrated RIBE clearly and precisely in experiments [7–10]. Some of the experiments indicate that the bystander effect is independent of dose, number of irradiated cells, and the linear energy transfer (LET) of the radiation [11, 12].

 Currently, it is presumed that the signal transmission from irradiated cells to unirradiated cells is realized via several molecules secreted by the irradiated cells, such as interleukins, growth factors, and nitric oxide [13–15]. Facoetti et al. [16] have measured Interleukin-8 (IL-8) and Interleukin-6 (IL-6), as candidates of the bystander signals, and evaluated the influence of experimental conditions (e.g., cell density and medium volume) on the presence and release of these molecules in the medium. On another front, the mechanism of RIBE focusing on the intercellular signal transmission has been investigated by simulation studies considering the geometry of *in vitro* experiments. Brenner et al. have proposed a mathematical model for RIBE, the Bystander and Direct (BaD) model, based on a binary phenomenon in a small sensitive subpopulation of cells and suggested that the bystander effect is important only at small doses [17]. Khvostunov and Nikjoo [18] have developed a biophysical model taking account of the bystander signal molecules with a certain probability of emission from the irradiated cells (bystander diffusion modeling: BSDM), and some researchers have simulated the RIBE in different ways such as a fully Monte Carlo procedure [19–24]. However, the underlying mechanisms of the molecules have not been clearly shown. The information on the number of original signals and the emitting probabilities of the signals from bystander cells is scarce, while the model itself still remains to be validated through a variety of means to examine this effect.

 In this study, we present a simulation model of RIBE focusing on the signal number, in which the behavior of the bystander signal molecules is analyzed with a Monte Carlo technique. The model involves the signal emission probability from the bystander cells following Poisson statistics. 

## 2. Methods and Modeling

 The current investigations are based on experiments and computer simulations to make clear the biological characteristics and reactive properties of RIBE. Recently, Xia et al. simulated RIBE with a Monte Carlo technique aimed at studying how bystander cells react at different distances from the irradiated cell [22]. However, the parameters were arbitrary in their model, making it difficult to determine them uniquely.

### 2.1. Modeling of RIBE with Signal-Emission Probability following Poisson Statistics

 Our simulation algorithm is constructed for demonstrating the clonogenic assays of V79 cells which were randomly seeded as described in Schettino et al. [25]. In Schettino's experiment, individual V79 cells were exposed to a focused carbon K-shell X-ray microbeam (278 eV) in the 0–2 Gy range. The V79 cells were randomly allocated in a circle with radius 3 mm in the dish corresponding to the experimental condition. The assumptions underlying this simulation are described as follows.

(1) At the beginning, the original bystander signals are generated by the irradiated cell and diffuse in cell culture medium through Brownian motion with a mean square displacement (*r*) of
  (1) 〈r2(t)〉=4Dt.
Here, *D* is the diffusion coefficient (constant) of the molecule considered as the bystander signal, and *t* is the time. In order to estimate the spread pattern of the bystander signals in the culture medium, the two-dimensional (2D) motion of the signal molecules is considered as in (1). The simulation is performed on the assumption that the signaling molecules are cytokines, such as IL-8 or IL-6, which have a mass of about 10 kDa. Thus, the diffusion coefficient of cytokine in the cell culture medium can be estimated to be around 10^8^ nm^2^s^−1^ [26], while the unit time step Δ*t* is set to be 1 s.
(2) When a signal comes in the sphere of 5 *μ*m radius about the center of an unirradiated cell, the unirradiated cell is transformed into a so-called “bystander cell” with a probability *P*_dam_.
(3) The bystander signal is annihilated in the above reaction, and the transformed bystander cell reemits a certain number (*k*) of bystander signals with a probability *P*_re_. In the present model, we assume this probability to follow the Poisson distribution. The probability *P*_re_ is given by
  (2) Pre(k)=μkk!e−μ.
Here, *μ* is the mean value of the signal number. Then, the total reemission probability, *P*_reT_, is deduced by the summation of *P*_re_(*k*) over *k* = 1 to infinity (the case *k* = 0 is excluded because this brings about no emission).
(4) All signals have a certain life time (60 hr) and disappear at that time.

The basic idea in this model is that the signal transmission between cells is made by mobile entities such as molecules (but the identity of them remains to be seen). The simulation is performed with a Monte Carlo technique, where 259200 steps are tracked for demonstrating 3 days in real experimental time. This procedure is repeated to achieve statistically satisfactory iterations for a variety of initial random seeds. The program code is written in FORTRAN90, and the Mersenne Twister is used as the random number generator. The cell damage probability *P*_dam_ = 0.01 is chosen in consideration of the fact that cytokine-specific receptors cover 1% of the cell surface [27]. Although Xia et al. presumed the life time of all signals to be 36 hours [22], we assumed the life time to be 60 hours since Mothersill and Seymour showed that bystander signals are still active at 60 hours after irradiation [6]. Furthermore, the number of original signals generated at the irradiated cell was set to be 10, 15, 20, 25, or 30. For each original signal number, the mean value of the reemitting signal number, **μ**, was varied from 0.5 to 5.0 for estimating the optimal number of the reemission signals.

## 3. Results and Discussion

**Figure 1:**
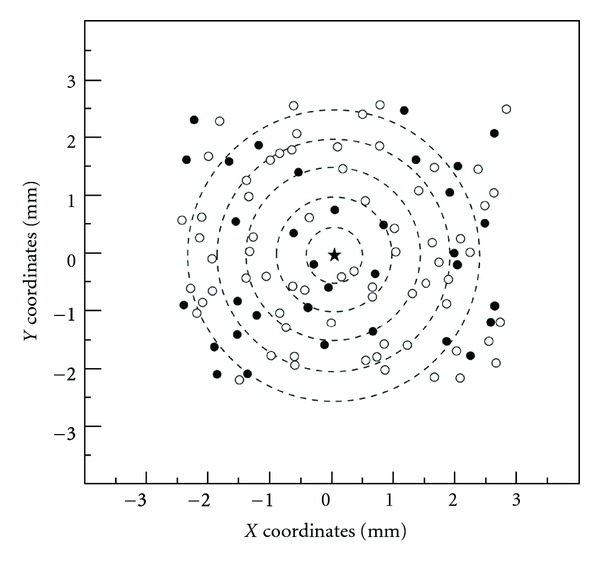
An example of the spatial distribution of cells in the simulation. The star is the position of the irradiated cell. Open circles represent the cells that remained to form healthy colonies, while filled circles are cells damaged by bystander responses. Dashed lines are for illustrative purposes only. Here, the number of original signals is 25, while the mean number of reemission signals is 1.5.

 Investigations using the microbeam technique have pointed out that the RIBE occurs independently of the distance from the irradiated cell, the absorbed dose, and the linear energy transfer (LET) of the ionizing radiation in their experimental conditions [11, 12]. According to Schettino et al., the fraction of damaged cells per annulus is statistically constant over the distance from the irradiated cell for the doses of 0.2 and 2 Gy. The results in the present model were compared with those for the actual fraction of bystander cells as a function of distance from the irradiated cell reported by Schettino et al. In recent studies by Ballarini et al. [20] and Xia et al. [22], normal cells were arrayed at a regular interval between cells in a grid configuration. In such a condition, the distances from the irradiated cell are distributed in a cyclic manner, yielding the signal transmission distance with a periodic pattern. This may lead to incorrect demonstrations for the experimental condition. After the bystander signal tracking in the simulation, the damaged bystander cells were counted, and their *X-Y* positions were recorded.  Figure 1 shows an example of cell distribution in the cell culture under a microbeam irradiation to the center cell represented as a star. The number of original signals is 15, while the mean number of reemission signals (**μ**) is 2.0. All living cell positions were acquired from the experiment by Schettino et al. Open and filled circles correspond to the healthy cells and the damaged bystander cells, respectively. One-hundred three cells in the experiment were entirely allocated on the dish in the simulation model. The dashed lines mark the virtual circular regions in 3 mm radius about the center of the irradiated cell.

 Each region delimited by adjacent dashed lines is defined as an “annulus.” The width of each annulus is set at 0.5 mm. Here, the number of original signals was set to be 25, and the mean number of reemission signals was 1.5. As shown in Figure 1, the damaged bystander cells are sparsely distributed in the cell culture medium, which is consistent with the results reported in previous studies [7, 12, 27].

**Figure 2:**
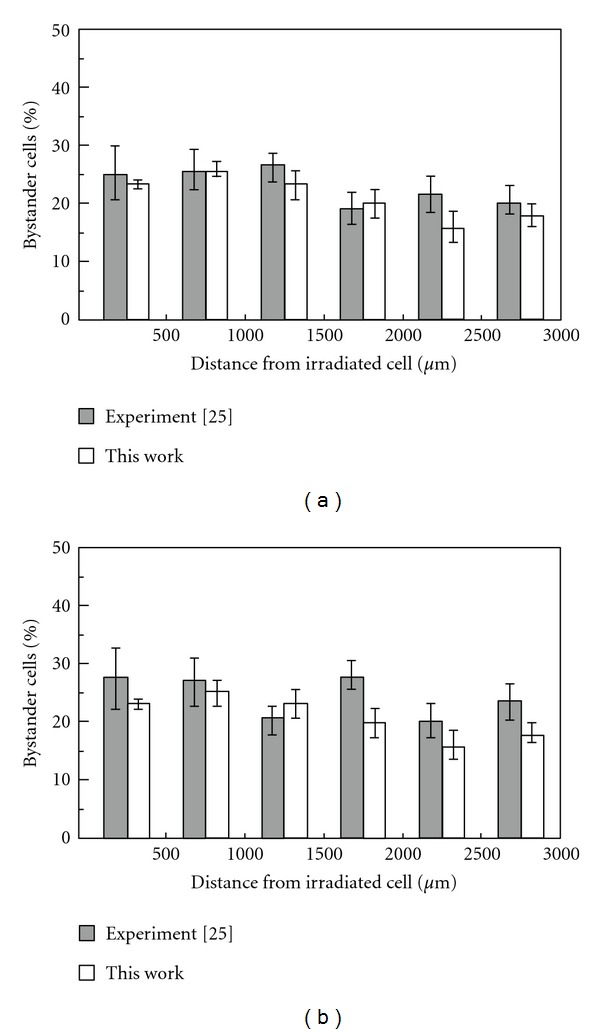
Ratio of the bystander cells as a function of distance from the irradiated cell. Gray column represents Experiment [25], white column represents simulation results: (a) for 0.2 Gy, single-cell irradiation, (b) for 2 Gy, single-cell irradiation. Here, the number of original signals is 15, and the mean number of reemission signals is 2.0.

 Figures 2(a) and 2(b) compare the proportion of bystander cells in each annulus in the experimental [25] and simulation results under 0.2 and 2 Gy X-ray irradiation. The simulation results for both cases were obtained with the number of original signals 15 and the mean number of reemission signals 2.0. The agreement between the simulation and experimental results is fairly good, except for the ratio in the fourth annulus from the center in the 2 Gy case. Many simulation trials, within the fluctuation of reemission probability used here, tell us that the ratio of bystander cells does not always decrease monotonically as the distance from the center increases. The uniform spatial distribution of the bystander cells appears presumably as a consequence of the signal transmission process with a reemission probability. As a test of coincidence for the ratios between the experiment and simulation, the root mean square difference (RMSD) was examined for every mean reemission signal number (**μ**). The formula of RMSD is given by.

(3) $$\begin{array}{c} \text{RMSD}\;\left(\%\right)=\sqrt{\frac{\sum_{i=1}^{N}{\left[r_{\exp}\left(i\right){-r}_{\text{sim}}\left(i\right)\right]}^{2}}{N}}. \end{array}$$

**Figure 3:**
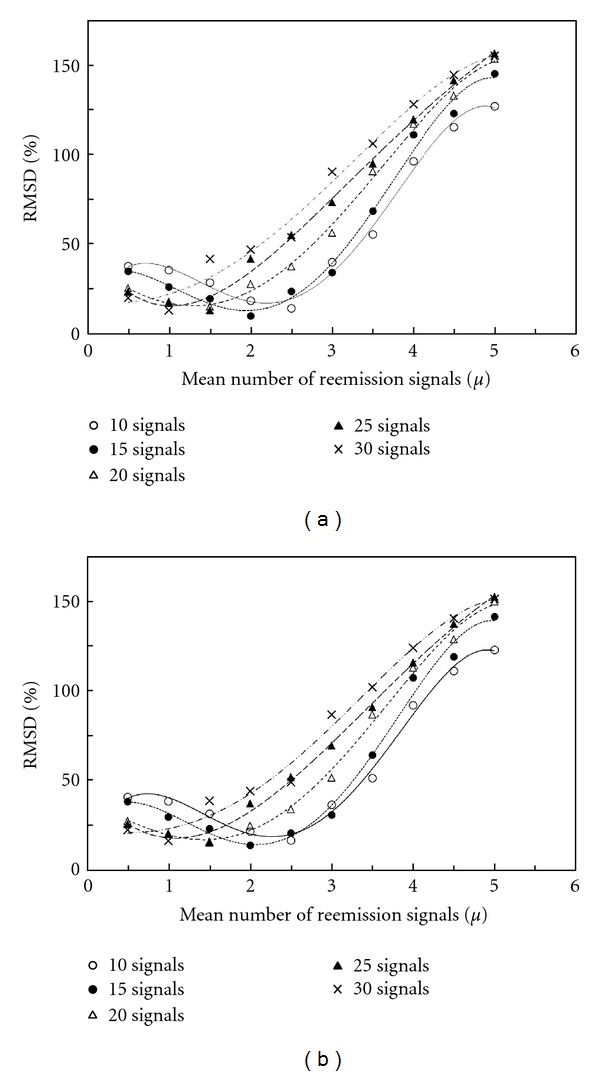
The RMSD between simulation and experimental results plotted against the mean number of reemission signals with best-fit curves. Each graph illustrates five cases for original signal number 10, 15, 20, 25, and 30: (a) for 0.2 Gy, (b) for 2 Gy.

Here, *r*_exp⁡_ and *r*_sim_ are the average rates of bystander cells per total cells in each annulus (%) obtained by the experiment and simulation, and *i* represents an annulus number (up to *N* = 6 in this study). The RMSD versus mean number of reemission signals is shown in Figures 3(a) and 3(b). Each graph illustrates the cases for original signal number of 10, 15, 20, 25, and 30. It should be noted that the RMSD has a minimum in the mean number of reemitted signals ranging from 1.0 to 2.5 in all cases. The result also suggests that the mean number at the minimum of RMSD is inclined to decrease with increasing original signal number.

 A salient feature of the present model is that the signals from bystander cells are treated with the reemission probabilities following Poisson statistics. It should be natural that the phenomenon of signal emission is stochastic, and thus, the number of released signals from bystander cells obeys Poisson statistics. The advantage of using the Poisson distribution is that the number of parameters for the simulation can be reduced because the distribution is defined by only one parameter (**μ**). Our simulation results show a fair agreement with the experiment in corresponding conditions, where the optimal probability is deduced through a large number of the simulation runs. As a result, we find that the most probable reemission probability (*P*_reT_) fall within 0.63–0.92 for the mean number of signals ranging from 1.0 to 2.5. This procedure to obtain the minimum of RMSD enables us to determine the signal reemission probability without arbitrary adjustments. A natural extension of the simulation would be to compute the time lapse of the signal transmission.

## 4. Conclusions

 In this study, the radiation-induced bystander effect (RIBE) was investigated by using a Monte Carlo simulation technique for a diffusion model of the bystander signals in comparison with the published experimental data. We have estimated the probable number of signals and reemission probability of signals from the bystander cells. The specific points of the model in this study are summarized below.

1. The model was constructed by introducing the bystander signal emission following Poisson statistics.
2. The simulation was able to reproduce the bystander cell spatial distribution featuring a uniform distribution in accord with the experiment.
3. The reemission signal number was deduced by comparing with the experimental result, where the root mean square difference (RMSD) between the simulation and experiment was found to have a minimum as a function of the mean reemission signal number.
4. The reemission probability of the signal was deduced in this simulation to range from 0.63 to 0.92, and the mean signal number is between 1.0 and 2.5.
